# Therapy and Outcomes of Patients with Relapsed Nonmetastatic Rhabdomyosarcoma: A Report from the French Society of Pediatric Oncology Malignant Mesenchymal Tumor Committee

**DOI:** 10.1002/cam4.70420

**Published:** 2024-11-29

**Authors:** François Sevrin, Emilie Bogart, Daniel Orbach, Marie Cécile Le Deley, Pablo Berlanga, Valérie Bernier, Nadège Corradini, Cindy Fayard, Florent Guerin, Sarah Jannier, Marie Karanian, Stéphanie Proust, Angélique Rome, Cécile Verité, Véronique Minard‐Colin, Anne‐Sophie Defachelles

**Affiliations:** ^1^ Department of Pediatric Oncology Oscar Lambret Center Lille France; ^2^ Department of Methodology and Biostatistics Oscar Lambret Center Lille France; ^3^ SIREDO Oncology Center (Care, Innovation and Research for Children, Adolescents and Young Adults With Cancer) PSL University, Institut Curie Paris France; ^4^ Department of Pediatric and Adolescent Oncology, Gustave‐Roussy Université Paris‐Saclay Villejuif France; ^5^ Department of Radiotherapy Institut de cancérologie de Lorraine Vandœuvre‐les‐Nancy France; ^6^ Institute of Pediatric Hematology and Oncology Léon Bérard Center Lyon France; ^7^ Imaging Department Oscar Lambret Center Lille France; ^8^ Department of Pediatric Surgery Université Paris‐Saclay, Assistance Publique Hôpitaux de Paris (AP‐HP), Bicêtre Hospital Le Kremlin‐Bicêtre France; ^9^ Pediatric Onco‐Hematology Unit University Hospital of Strasbourg Strasbourg France; ^10^ Department of Pathology Léon Bérard Center Lyon France; ^11^ Department of Pediatric and Adolescent Hematogy and Oncology CHU Angers Angers France; ^12^ Department of Pediatric Oncology Timone Children's Hospital Marseille France; ^13^ Department of Pediatric and Adolescent Hematogy and Oncology Pellegrin Hospital Bordeaux France

**Keywords:** outcome, prognosis, relapse, rhabdomyosarcoma, salvage therapy, second‐line therapy

## Abstract

**Background:**

The prognosis for patients with relapse of localized rhabdomyosarcoma (RMS) remains poor, with limited evidence for optimal second‐line therapy. This study describes the management and outcomes of relapsed RMS patients in France.

**Methods:**

We retrospectively reviewed all nonmetastatic RMS patients enrolled in France in the RMS 2005 study who relapsed between 2006 and 2019 after achieving complete local control, defined as complete remission or stable residue ≥ 6 months after treatment completion. Data were extracted from the RMS 2005 database and medical records.

**Results:**

Ninety‐five patients relapsed at a median age of 6.0 years (range: 1.0–27.0). The median time from diagnosis to relapse was 17.5 months (range: 7.4–82.0). Most patients had embryonal RMS (65.3%) and local/locoregional relapses (71.6%). The first relapse treatment included chemotherapy (all except two patients), radiotherapy (52.6%), and surgery (48.4%). Second‐line chemotherapy yielded a 58.5% objective response rate after 3 ± 1 cycles. Fifty‐five patients achieved second complete remission. With a median follow‐up of 7.2 years from the first relapse (range: 0.3–11.3), 5‐year progression‐free survival was 26% (95% CI: 18–36), and 5‐year overall survival was 35% (95% CI: 25–45). Importantly, no patient survived relapse without receiving locoregional treatment (surgery and/or radiotherapy).

**Conclusion:**

This study confirmed the inconsistencies in therapy and the poor prognosis for relapsed RMS but highlighted the potential for long‐term survival in patients who received surgery and/or radiotherapy, emphasizing the crucial role of achieving local control in improving outcomes at relapse.

**Abbreviations**
95% CI: 95% confidence interval
CR: complete response
EpSSG: European pediatric Soft Tissue Sarcoma Study Group
ORR: objective response rate
OS: overall survival
PD: progressive disease
PFS: progression‐free survival
PR: partial response
RMS: rhabdomyosarcoma
RT: radiotherapy
SIOP‐MMT: Malignant Mesenchymal Tumor Group of the International Society of Pediatric Oncology
VC: vinorelbine–cyclophosphamide
VI: vincristine–irinotecan
VIT: vincristine–irinotecan–temozolomide

## 1 Introduction

Rhabdomyosarcoma (RMS) is the most common soft tissue sarcoma in children, representing approximately 4% of all childhood cancers [1]. Despite multimodal therapies achieving long‐term survival rates > 70% for patients with localized disease, 25%–30% still experience recurrence [2, 3, 4]. Outcomes for these patients are poor, with overall survival (OS) around 30% [3, 4]. Possible approaches to relapse include intensive multi‐agent chemotherapy with local therapy, depending on surgical accessibility and prior radiotherapy (RT), and enrollment in phase I/II trials with new agents [5, 6, 7, 8, 9]. However, these trials typically do not collect data on local treatment and therapy following subsequent relapses, neglecting potentially crucial information for refining salvage strategies.

Some studies have explored the prognostic implications of various factors in relapsed RMS [3, 4, 10, 11, 12, 13]. Chisholm et al., on behalf of the Malignant Mesenchymal Tumor group of the International Society of Pediatric Oncology (SIOP‐MMT), developed a nomogram‐based prognostic score, considering multiple risk factors to assess salvage potential after relapse [3]. Although this nomogram aids physicians in deciding between salvage therapy with curative intent and experimental or palliative therapy for children with a poor prognosis, no internationally agreed standard of care exists for relapsed RMS.

This retrospective study involved 95 patients in France diagnosed with nonmetastatic RMS who relapsed after first‐line treatment. This study aimed to describe the management and outcomes of this nationwide cohort and try to better define the optimal salvage strategy. Moreover, it validated the prognostic value of the nomogram established by the SIOP‐MMT group in an independent cohort.

## 2 Materials and Methods

We retrospectively reviewed all patients with nonmetastatic RMS prospectively enrolled in France in the RMS 2005 study (NCT00379457) between November 2005 and December 2016 who experienced relapse after achieving complete local control, defined as complete remission or stable residue ≥ 6 months after treatment completion. All relapses between January 2006 and December 2019 were included, excluding cases without available data. Any event other than relapse was excluded. Ethical approval and parent/patient consent were obtained during the protocol inclusion.

Patients in the RMS 2005 study were newly diagnosed with pathologically confirmed localized RMS and aged 6 months–21 years. Each patient was assigned to a specific subgroup according to the European pediatric Soft tissue sarcoma Study Group (EpSSG) stratification system. Treatment followed subgroup‐specific recommendations, including chemotherapy, local therapy (surgery and/or RT), and potential 6‐month maintenance therapy with vinorelbine‐cyclophosphamide (VC) [14, 15, 16].

Upon relapse, a comprehensive clinical and radiological assessment of disease staging was recommended [15, 16]. Disease assessment could include computed tomography (CT) scan or magnetic resonance imaging of the site of relapse, evaluation of lymph node involvement (confirmed by histology when necessary), chest CT‐scan, 99mTechnecium scan or 18‐FDG PET CT and bone marrow evaluation. Pathological confirmation of relapse, including molecular profiling, was advised, notably through the precision medicine trial MAPPYACTS (NCT02613962) from February 2016 [17]. Physicians had discretion in selecting the chemotherapy regimen upon relapse, though the EpSSG recommended enrolling these patients in the phase II VIT trial (NCT01355445) during the opening phase between March 2012 and April 2018 [8]. Surgery and/or RT were recommended for relapse sites whenever feasible.

Data from the RMS 2005 study database, collected prospectively, were supplemented by retrospectively extracting data from patient medical records regarding first and subsequent relapses. Treatment response followed the three‐dimensional World Health Organization response criteria for the primary lesion and RECIST 1.1 criteria for metastatic sites [18, 19]. Objective response rate (ORR) was defined as the proportion of patients with a complete response (CR) or partial response (PR, defined as at least a 50% reduction in the volume of the primary lesion according to WHO response criteria, and at least a 30% reduction in the sum of the diameters of target lesions in the case of metastatic disease, according to RECIST 1.1 criteria). Tumor response assessment relied on medical record data; no retrospective central imaging review was performed. A case‐by‐case retrospective review of RT fields was conducted for patients re‐irradiated with curative intent for in‐field recurrence. Maintenance therapy at relapse was defined as the initiation of chemotherapy with VC, VI (vincristine‐irinotecan), or VIT (vincristine‐irinotecan‐temozolomide) following the achievement of a complete response after second‐line treatment, or as prolonged chemotherapy with VC, VI, or VIT for a total duration of 12 or more cycles. The chance of cure was estimated for each patient using the nomogram developed by the SIOP‐MMT [3]. For this analysis, chemotherapy regimens at initial diagnosis were classified into two categories: < 4 drugs and ≥ 4 drugs. The < 4 drugs group included regimens with vincristine and dactinomycin (VA) only; ifosfamide, vincristine, and dactinomycin (IVA); and vincristine, dactinomycin, and cyclophosphamide (VAC). The ≥ 4 drugs group comprised all other regimens, including patients receiving ifosfamide, vincristine, dactinomycin, and doxorubicin (IVADo), as well as those who received maintenance chemotherapy with VC following induction chemotherapy.

Comparison of early (within the first 18 months from initial diagnosis) and late relapses included Student's *t*‐tests for quantitative variables and chi‐squared or Fisher's exact tests for qualitative variables. Median follow‐up was estimated using the reverse Kaplan–Meier method. OS and progression‐free survival (PFS) curves were estimated using the Kaplan–Meier method from the date of relapse. Multivariate Cox models were employed to study prognostic factors for post‐relapse OS, after selecting based on univariate analyses (factors associated with a *p*‐value < 0.20 in univariate analyses). The significance threshold for the multivariate analyses was set at 0.05. We considered the prognostic score defined by the nomogram developed by the SIOP‐MMT group and estimated Harrell's *C*‐index to assess its prognostic accuracy [3]. Analyses were performed using Stata software version 15.0 (StataCorp. 2017. Stata Statistical Software: Release 15. College Station, TX: StataCorp LLC).

## 3 Results

In total, 497 French patients were identified in the RMS 2005 database (Figure S1). Complete local control was achieved in 452 patients, of whom 96 (21.2%) experienced relapse. Excluding one patient with unavailable relapse data, the study population comprised 95 patients. Patient characteristics and details of the first‐line treatment are presented in Table 1. The median time from initial diagnosis to first relapse was 17.5 months (range: 7.4–82.0) (Table 2). Fifty‐three patients (55.8%) had local relapse only, 13 (13.7%) had relapse involving regional lymph nodes only, two (2.1%) had locoregional relapse involving both the primary site and regional lymph nodes, 15 (15.8%) had metastatic relapse only, and 12 (12.6%) had combined relapse. Among the 27 patients with metastatic relapse, 17 (63.0%) had a single, five (18.5%) had two, and five (18.5%) had at least three metastatic sites. Among the 68 patients with local/locoregional relapse, 49 received prior irradiation, and 37 experienced in‐field recurrence. Relapse was confirmed pathologically in 68 patients (71.6%). Molecular tumor profiling was conducted in 11 patients (11.6%), with seven enrolled in the MAPPYACTS trial.

**TABLE 1:** Patient, disease, and treatment characteristics at initial diagnosis.

Characteristics (*n* = 95)	*n*	%
Patients		
Age at initial diagnosis		
< 1 year	7	7.4%
1–9 years	58	61.1%
≥ 10 years	30	31.6%
Sex		
Male	59	62.1%
Female	36	37.9%
Initial tumor characteristics		
Histology		
Non‐alveolar RMS	62	65.3%
*Embryonal RMS*	58	61.0%
*Spindle cell RMS*	3	3.2%
*Not otherwise specified*	1	1.1
Alveolar RMS	33	34.7%
Detection of a FOXO1 fusion transcript		
No	41	43.1%
Yes	24	25.3%
Not available	30	31.6%
Site of primary tumor		
Favorable	31	32.6%
*Genitourinary non‐bladder prostate*	12	12.6%
*Orbit*	10	10.5%
*Head neck non‐parameningeal*	9	9.5%
Unfavorable	64	67.4%
*Parameningeal*	29	30.5%
*Extremities*	14	14.7%
*Other site*	14	14.7%
*Bladder–prostate*	7	7.4%
Tumor size		
≤ 5 cm	43	45.3%
> 5 cm	52	54.7%
Lymph nodes		
N0	69	72.6%
N1	24	25.3%
NX	2	2.1%
IRS group: post‐surgical staging		
IRS Group I	4	4.2%
IRS Group II	12	12.6%
IRS Group III	79	83.2%
Stratification group		
Low risk, subgroup A	1	1.1%
Standard risk	31	32.7%
*Subgroup B*	2	2.1%
*Subgroup C*	20	21.1%
*Subgroup D*	9	9.5%
High risk	50	52.6%
*Subgroup E*	20	21.1%
*Subgroup F*	11	11.6%
*Subgroup G*	19	20.0%
Very high risk, subgroup H	13	13,7%
First‐line treatment	95	100%
Chemotherapy regimen		
Vincristine and D‐actinomycin	1	1.1%
Alkylating‐based chemotherapy (without doxorubicin)	60	63.2%
Doxorubicin‐based chemotherapy	34	35.8%
Maintenance therapy: Yes	35	36.8%
Number of chemotherapy drugs		
2 (VA)	1	1.1%
3 (IVA/VAC)	48	50.5%
≥ 4 (IVADo ± maintenance therapy or IVA + maintenance therapy)	46	48.4%
Radiotherapy: Yes	72	75.8%

Abbreviations: IRS, Intergroup Rhabdomyosarcoma Study; IVA, ifosfamide, vincristine, and d‐actinomycin; IVADo, ifosfamide, vincristine, d‐actinomycin, and doxorubicin; RMS, rhabdomyosarcoma; VA, vincristine and d‐actinomycin; VAC, vincristine, d‐actinomycin and cyclophosphamide.

**TABLE 2:** Characteristics of the first relapse according to the time interval from the initial diagnosis to the first relapse.

Characteristics	1st relapse < 18 months from the initial diagnosis; *n* = 49		1st relapse ≥ 18 months from the initial diagnosis; *n* = 46		Overall; *n* = 95		*p* ^b^
Age at first relapse (years)							
Median (range)	6.0	(1.0–22.0)	8.0	(2.0–27.0)	6.0	(1.0–27.0)	
1–9 years	32	65.3%	26	56.5%	58	61.1%	0.38
≥ 10 years	17	34.7%	20	43.5%	37	38.9%	
Time interval from the initial diagnosis to the 1st relapse (months)							
Median (range)	12.4	(7.4–17.9)	23.6	(18.0–82.0)	17.5	(7.4–82.0)	
Relapse > 5 years from the initial diagnosis	0		2	4.3%	2	2.1%	
Patient previously treated with radiotherapy	38	77.6%	34	73.9%	72	75.8%	
If yes, relapse location							
Outside of the RT fields	15	39.5%	11	32.4%	26	36.1%	
In the RT field	20	52.6%	23	67.6%	43	59.7%	
Unknown	3	7.9%	0	0%	3	4.2%	
Pathological confirmation at relapse	28	57.1%	40	87.0%	68	71.6%	
Molecular profiling performed	4		7		11	11.6	
Type of recurrence							0.39
Local/locoregional only	35	71.4%	33	71.7%	68	71.6%	
Metastatic only	6	12.2%	9	19.6%	15	15.8%	
Combined	8	16.3%	4	8.7%	12	12.6%	
Detail of local/locoregional recurrences, including combined recurrences (*n* = 80)	43		37		80		
Local only	28	65.1%	27	73.0%	55	68.8%	
Regional lymph nodes only	10	23.3%	9	24.3%	19	23.8%	
Local with regional lymph nodes	5	11.6%	1	2.7%	6	7.5%	
Detail of metastatic sites of recurrence, including combined recurrences (*n* = 27)^a^	14		13		27		
Distant lymph node	6		6		12		
Lung	4		6		10		
Bone and/or bone marrow	3		3		6		
Subcutaneous	2		4		6		
Central nervous system	2		0		2		
Other site(s)	4		6		10		

Abbreviations: *N*, number; RT, radiotherapy.

^a^ The same patient may have several sites of metastases.

^b^ Student's t‐test for quantitative variables and chi‐squared tests for qualitative variables.

Following disease recurrence, two patients (2.1%) with locoregional recurrence received no anti‐cancer treatment because of rapid tumor progression and early death. The remaining 93 patients received various second‐line chemotherapy regimens (Table S1). Most patients (53.7%) received anthracycline‐based chemotherapy prior to the initiation of the phase 2 VIT trial, whereas 69.2% received irinotecan‐based chemotherapy after the trial opened. Notably, of the 41 patients who relapsed during the VIT trial enrolment period, 23 (56.1%) were ultimately enrolled in the protocol. Overall, the first tumor response evaluation to chemotherapy was conducted after 1 cycle for 1 patient (initially scheduled after 2 cycles but brought forward due to clinical suspicion of disease progression), after 2 cycles for 64 patients, after 3 cycles for 13 patients, and after 4 cycles for 4 patients. It showed an ORR of 58.5% with three CR (3.7%) and 45 PR (54.9%); 14 patients (17.1%) had progressive disease (PD). Responses to each chemotherapy regimen group are presented in Table S2. The ORR at 3 ± 1 cycles was 50% for early relapses and 65% for late relapses.

### 3.1 Treatment at First Relapse—Nonmetastatic Relapses

All 66 patients with nonmetastatic relapses received second‐line chemotherapy, with a 50.9% ORR at 3 ± 1 cycles (Table 3).

**TABLE 3:** Treatments at time of first relapse.

Characteristics of treatments at first relapse	Non metastatic; *n* = 66^a^		Metastatic; *n* = 27		Overall; *n* = 93^a^	
Second‐line chemotherapy	66	100%	27	100%	93	100%

Chemotherapy regimen						
Anthracycline‐based	27	40.9%	6	22.2%	33	35.5%
Irinotecan‐based	24	36.4%	17	63.0	41	44.1%
Including VI or VIT regimens	23	34.8%	15	55.6%	38	40.9%
Alkylating agent‐based without irinotecan	3	4.5%	1	3.7%	4	4.3%
Platinum‐based	5	7.6%	2	7.4%	7	7.5%
Low‐dose chemotherapy	7	10.6%	1	3.7%	8	8.6%
**Number of received cycles: median (range)**	4.0	(1.0–14.0)	6.0	(1.0–14.0)	6.0	(1.0–14.0)

Tumor response after 3 ± 1 cycles (*n* = 82, MD = 9, NA = 2^b^)						
Response	29/57	50.9%	19/25	76.0%	48/82	58.5%
CR	3		0		3	
PR	26		19		45	
No response	28/57	49.1%	6/25	24.0%	34/82	41.5%
SD	15		5		20	
PD	13		1		14	
^c^ **or PD**	25	37.9%	13	48.1%	38	40.9%
Focal treatment during second‐line chemotherapy^d^	43	65.2%	15	55.6%	58	62.4%
**Local treatment**	43	65.2%	4/12	33.3%^f^	47/78	60.3%^f^
Exclusive surgery	14	21.2%	1	8.3%	15	19.2%
Exclusive radiotherapy	11	16.7%	3	25%	14	17.9%
Both	18	27.3%	0	0%	18	23.1%
Site of surgery^e^						
Primary tumor	23		0		23	
Regional lymph node area	10		1		11	
Quality of surgical resection (*n* = 33)						
R0	18	56.3%	0	0%	18	54.5%
R1	12	37.5%	1	100%	13	39.4%
R2	2	6.3%	0	0%	2	6.1%

Radiation sites^e^						
Primary tumor	19		0		19	
Lymph node	11		3		14	
Radiation dose in Gy: median (range)	50.4	(15.0–60.0)	50.4	(12.6–50.4)	50.4	(12.6–60.0)
**Focal treatment of distant metastases**	NA		15/27	55.6%	15/27	55.6%^g^
Exclusive surgery			2		2	
Exclusive radiotherapy			7		7	
Both			6		6	

Site of surgery^e^						
Central nervous system			2		2	
Lung			1		1	
Liver			1		1	
Other site			4		4	

Quality of resection						
R0			3		3	
R1			4		4	
R2			1		1	

Radiation sites^e^						
Bone			4		4	
Lung			3		3	
Distant lymph node			3		3	
Central nervous system			2		2	
Liver			1		1	
Subcutaneous			1		1	
Other site			1		1	
Radiation dose in Gy: median (range)			49.5	(30.0–54.0)	49.5	(30.0–54.0)
**Achievement of 2nd complete remission**	41	62.1%	9	33.3%	50	53.8%
PD before any local treatment	23	34.8%	11	40.7%	34	36.6%
2nd complete remission after further treatment	4		1		5	

Achievement of 2nd complete remission						
No	21	31.8%	17	63.0%	38	40.9%
Yes	45	68.2%	10	37.0%	55	59.1%

For patient achieving 2nd complete remission only:						
Treatment received:						
Chemotherapy	45		10		55	
Exclusive surgery	12		2		14	
Exclusive radiotherapy	11		2		13	
Surgery and radiotherapy	22		5		27	
Surgery considered as mutilating^h^	12		0		12	
Maintenance therapy	24	36.4%	7	25.9%	31	33.3%
Persistent 2nd complete remission at treatment completion	37	56.1%	9	33.3%	46	49.5%

Abbreviations: CR, complete response; Gy, gray; MD, missing data; *N*, number; NA, not applicable; PD, progressive disease; PR, partial response; R0, negative margins; R1, microscopically positive margins; R2, gross residual disease left behind; SD, stable disease.

^a^ Among the 95 analyzed patients, two with locoregional relapse received no anti‐cancer treatment due to early death and were not included in the analysis.

^b^ The two patients who underwent immediate surgery with grossly complete resection were not included in the analysis.

^c^ Change of chemotherapy while the patient had no progressive disease.

^d^ Focusing solely on focal treatment received during second‐line chemotherapy, excluding focal treatment received during subsequent lines of chemotherapy following disease progression.

^e^ A same patient can have several sites of surgery and/or radiotherapy.

^f^ Metastatic relapses without local/locoregional relapse were not included in the analysis.

^g^ The analysis included only metastatic relapses.

^h^ Surgery was considered mutilating when it led to significant long‐term anatomical, functional, or cosmetic impairments.

Forty‐three patients (65.2%) underwent local treatment during second‐line chemotherapy, including exclusive surgery in 14 (all had received prior RT at initial diagnosis), exclusive RT in 11, and a combination of both in 18.

Of the 23 patients experiencing PD during second‐line chemotherapy before any local treatment, four achieved a second complete remission with subsequent chemotherapy and local treatment (surgery and RT for all of them).

In total, 36 patients underwent surgery. The quality of resection was R0 (negative margins) in 20 patients, R1 (microscopically positive margins) in 14, and R2 (grossly positive margins) in two, resulting in a second complete remission in 34 patients. Surgery was considered mutilating (leading to significant long‐term anatomical, functional, or cosmetic impairments) in 12 patients.

In total, 33 patients received curative‐intent RT: 17/19 (89%) without prior RT, 16/47 (34%) with prior RT, including eight who underwent re‐irradiation partially involving the primary field—five with external beam radiation therapy and three with proton therapy. Re‐irradiation followed surgery in 7/8 cases, and therefore targeted a restricted area. Median doses were 50.4 Gy (range: 50.4–55.8) at diagnosis and 50.4 Gy (range: 40.0–54.0) at relapse, with a median interval of 2.2 years (range: 1.1–4.2) between the 2 irradiations.

Overall, 45/66 patients (68.2%) achieved a second complete remission, with 24 receiving maintenance therapy (19 VC, three VI, and two VIT regimens) for a median of 10.0 months (range: 0.9–12.8). Thirty‐seven maintained remission at treatment end, and 29 were alive at a median follow‐up of 7.2 years (range: 1.0–11.3) post‐first relapse, including three of four patients with prior PD during second‐line therapy. A key finding was that no patient survived relapse without undergoing locoregional treatment (surgery and/or RT).

### 3.2 Treatment at First Relapse—Metastatic Relapses

All 27 patients with metastatic recurrence received second‐line chemotherapy (Table 3). A 76% ORR was observed at 3 ± 1 cycles. One patient achieved a second remission exclusively through chemotherapy. Fifteen patients (55.6%) underwent local treatment of the primary and/or metastatic sites during second‐line chemotherapy, and eight achieved a second complete remission.

Of the 11 patients with PD before any local treatment, one ultimately achieved a second complete remission with subsequent therapy.

Overall, 10/27 patients with metastatic relapse (37.0%) achieved a second complete remission, and six (22.2%) remained alive at a median follow‐up of 3.7 years (range: 0.3–8.7). Among these six patients, all but one had a follow‐up of more than 3 years after the first relapse, and all but one had a single metastatic site at the time of first relapse; four were disease‐free at the time of the latest follow‐up.

### 3.3 Outcomes

Among the 95 patients with a first relapse, 55 achieved a second complete remission. Of these, 25 experienced a second relapse after a median of 17 months (range: 3–42) from the first relapse; seven experienced a third relapse, and one experienced > 3 relapses (Table 4). Five of the 25 patients (20%) with subsequent relapses remained alive at a median follow‐up of 5.4 years (range: 3.2–11.1) after the first relapse. Overall, 35 patients remained alive at a median follow‐up of 7.2 years (range: 0.3–11.3) from the first relapse, with 29 of them having a follow‐up duration exceeding 3 years. Of these 35 patients, 29 were in second complete remission, three in third complete remission, and one in fourth complete remission at the last follow‐up. One patient was still on treatment for a second relapse 3.5 years after the first relapse. The last patient with metastatic PD was lost to follow‐up 4 months after the first relapse. Two patients developed secondary malignancies without a known cancer predisposition syndrome: one developed undifferentiated pleomorphic sarcoma in the RT field and another developed glioblastoma. Five‐year PFS and OS rates were 26% (95% confidence interval [CI]: 18–36) and 35% (95% CI: 25–45), respectively.

**TABLE 4:** Overall characteristics of all relapses.

Characteristics of relapse	First relapse; *n* = 95		Second relapse; *n* = 25		Third relapse; *n* = 7	
Site						
Local/locoregional only	68	71.6%	15	60.0%	4	57.1%
Metastatic ± local	27	28.4%	10	40.0%	3	42.9%
Patient previously treated with radiotherapy	72	75.8%	24	96.0%	7	100%
Relapse within radiotherapy field						
Yes	44	46.3%	16	64.0%	6	85.7%
No	48	50.5%	6	24.0%	1	14.3%
Unknown	3	3.2%	3	12.0%	0	0%
Treatment^a^						
Systemic therapy (± local therapy)	93	97.9%	23	92.0%	7	100%
Chemotherapy	93	97.9%	21	84.0%	7	100%
Immunotherapy						
Nivolumab	1		1		0	
Targeted therapy						
mTOR inhibitors	2		2		0	
Receptor tyrosine kinase inhibitors	0		3		0	
Other	1		3		1	
Non‐systemic therapy	65	68.4%	19	76.0%	4	57.1%
Surgery	46	48.4%	10	40.0%	2	28.6%
Radiotherapy	50	52.6%	14	56.0%	2	28.6%
Complete remission achieved	55	57.9%	10	40.0%	2	28.6%
Outcome						
Death	40	42.1%	14	56.0%	6	85.7%
Alive > 3 years from the last relapse	29^b^	32.6%	3^c^	13.0%	0^d^	
With disease	1^b^	1.1%	0^c^		0^d^	
Without disease	28^b^	31.5%	3^c^	13.0%	0^d^	

Abbreviation: *N*, number.

^a^ Including all treatments received during the same relapse due to tumor progression.

^b^ Six patients who were alive at the last follow‐up were not included in the analysis because their duration of follow‐up after the first relapse was less than 3 years.

^c^ Two patients who were alive at the last follow‐up were not included in the analysis because their duration of follow‐up after the second relapse was less than 3 years.

^d^ One patient who was alive at the last follow‐up was not included in the analysis because her duration of follow‐up after the third relapse was 2.7 years, which is less than 3 years.

**FIGURE 1:**
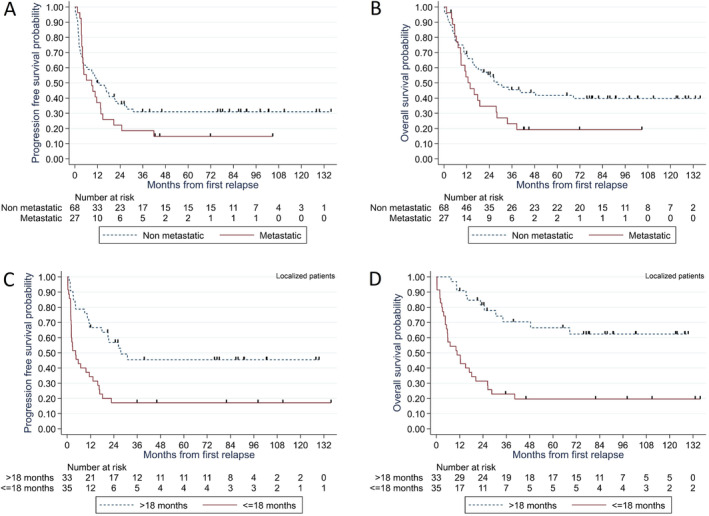
Kaplan–Meier survival curves. Progression‐free survival (PFS) (A) and overall survival (OS) (B) from the first relapse in all 95 patients included in the study according to the type of recurrence. PFS (C) and OS (D) from the first relapse in 68 patients with nonmetastatic relapse according to the time interval to relapse.

In patients with nonmetastatic relapse, the 6‐month PFS was 60% (95% CI: 48–71); 5‐year PFS and OS were 31% (95% CI: 20–42) and 42% (95% CI: 30–54), respectively (Figure 1A,B). Patients with nonmetastatic early relapse exhibited significantly poorer outcomes compared to late relapse regarding PFS (hazard ratio [HR] = 2.67, 95% CI: 1.46–4.90, *p* = 0.001) and OS (HR = 4.39, 95% CI: 2.17–8.88, *p* < 0.001; Figure 1C,D). The 5‐year OS rates were 70% (95% CI: 45%–85%) for patients with nonmetastatic relapse undergoing R0 surgical resection and 52% (95% CI: 22%–75%) for those with R1 surgical resection (Figure S2). The two patients with R2 resection died of PD. The 5‐year OS rates were 60% (95% CI: 37–77) for the 24 patients with nonmetastatic relapse receiving maintenance therapy and 68% (95% CI: 42–84) for those without maintenance therapy (Figure S3).

Of the 12 patients who underwent salvage mutilating surgery, 7 achieved a second complete remission and long‐term survival. This included 4 patients with extensive facial surgeries (2 with extended exenteration), 1 patient with lower limb amputation, 1 patient who underwent colpectomy, and 1 patient with severe genitourinary sequelae following total cystectomy with total urethrectomy and a sigmoid reservoir.

In multivariate analysis, four independent factors significantly increased death risk: unfavorable primary tumor site at diagnosis (*p* = 0.029), ≥ 4 drugs treatment at diagnosis (*p* = 0.005), prior RT (*p* < 0.001), and early relapse (*p* < 0.001) (Table 5). No significant associations were found for the histological subtype or the presence of the FOXO1 fusion transcript (*p* = 0.97 and *p* = 0.38, respectively). The mean probability of cure estimated by the nomogram developed by the SIOP‐MMT group was 0.28 ± 0.26 (range: 0.01–0.82). An increase in the prognostic score significantly reduced death risk (HR for a 0.1‐increase = 0.69, 95% CI: 0.60–0.81, *p* < 0.001). Harrell's C‐index for the predictive model was 0.69.

**TABLE 5:** Univariate and multivariate analysis of prognostic factors identified by Chisholm et al. after the first relapse.

Patient characteristics (*n* = 95)	Patients	Deaths	Univariate Cox model		Multivariate Cox model^a^	
			HR (95% CI)	*p*	HR (95% CI)	*p*
Initial diagnosis						
Histological subtype^b^				0.97		
Non alveolar	62	38	1		—	
Alveolar	33	22	0.99 (0.59–1.68)			
FOXO1 fusion transcript (MD = 21)^c^				0.38		
No	47	27	1		—	
Yes	27	19	1.30 (0.72–2.35)			
Primary tumor size^b^				0.002		0.13
≤ 5 cm	43	20	1		—	
> 5 cm	52	40	2.36 (1.38–4.06)			
Site of primary tumor^b^				< 0.001		0.029
Favorable	31	9	1		1	
Unfavorable	64	51	4.47 (2.20–9.12)		2.29 (1.09–4.82)	
Lymph nodes at initial diagnosis^b^				0.025		0.41
N0	69	39	1		—	
N1	24	20	2.11 (1.23–3.66)			
NX	2	1	0.91 (0.13–6.66)			
Initial IRS grouping				0.30	—	
Group I	4	2	1			
Group II	12	5	1.01 (0.20–5.22)			
Group III	79	53	1.89 (0.46–7.77)			
Prior first‐line chemotherapy^b^				0.002		0.005
< 4 drugs (VA, IVA, VAC)	49	24	1		1	
≥ 4 drugs (IVADo ± VC or IVA + VC)	46	36	2.28 (1.35–3.83)		1.20 (1.27–3.80)	
Prior radiotherapy^b^				0.001		< 0.001
No	23	7	1		1	
Yes	72	53	3.86 (1.75–8.50)		4.62 (2.01–10.6)	

First relapse						
Time between initial diagnosis and first relapse^b^				< 0.001		< 0.001
≥ 18 months	46	19	1		1	
< 18 months	49	41	4.28 (2.47–7.44)		6.18 (3.36–11.36)	
Metastatic recurrence^b^				0.053		0.09
No	68	39	1		—	
Yes	27	21	1.70 (0.99–2.90)			

Abbreviations: 95% CI, 95% confidence interval; HR, hazard ratio; IRS, Intergroup Rhabdomyosarcoma Study; IVA, ifosfamide, vincristine, and d‐actinomycin; IVADo, ifosfamide, vincristine, d‐actinomycin, and doxorubicin; MD, missing data; *N*, number; VA, vincristine and d‐actinomycin; VAC, vincristine, d‐actinomycin and cyclophosphamide; VC, vinorelbine and cyclophosphamide.

^a^ The final Cox model included the following variables: site of primary tumor; prior first‐line chemotherapy; prior radiotherapy; and time between initial diagnosis and first relapse. The other factors were not significantly associated with the risk of death when adjusted for these four variables.

^b^ Risk factors included in the predictive model of Chisholm et al.

^c^ Data obtained from both the initial diagnosis and first relapse.

## 4 Discussion

Based on a comprehensive data collection approach, this study retrospectively analyzed the clinical features, treatments, and outcomes of a large national series of 95 consecutive patients initially enrolled in the RMS 2005 study with localized RMS who relapsed after first‐line treatment.

Our dataset confirms the poor prognosis of relapsed RMS, including localized cases, with a 5‐year PFS of 26% and 5‐year OS of 35%, indicating limited progress over the past 30 years [3, 4, 10, 11, 12, 20, 21, 22, 23]. However, despite figures similar to those previously reported by Chisholm et al., our data were obtained from a cohort that underwent more extensive pretreatment, with a higher proportion receiving frontline RT [3]. Interestingly, our study also suggests the possibility, albeit limited, of prolonged survival even after second and third relapses, as well as in cases of metastatic recurrence. Furthermore, it suggests the potential for extended survival in patients who experience PD during second‐line therapy and subsequently undergo salvaged third‐line chemotherapy.

Although long‐term sequelae and quality of life were not formally analyzed in our study, it is important to mention that these factors should not be overlooked in long‐term survivors, especially since several of them underwent mutilating salvage surgery or re‐irradiation.

Our study revealed a 58.5% ORR with different second‐line chemotherapy regimens, confirming that RMS remains sensitive to chemotherapy even after relapse. The lack of a marked difference in chemosensitivity between early and late relapses, despite significant differences in outcomes, suggests that the chemotherapy response may not be a reliable predictor of PFS [13, 24, 25]. Consequently, PFS likely represents a more suitable primary endpoint than tumor response in phase II clinical trials.

Our findings underscore the critical importance of achieving complete local disease control. All patients with nonmetastatic relapse receiving no locoregional treatment (surgery and/or RT) died of PD. Although the limited size of our cohort did not allow for conclusive results, our data seem to underscore the importance of the quality of surgical resection at recurrence and align with prior research, emphasizing salvage surgery's significance [13, 22, 26]. Despite refraining from comparative statistical analysis due to the retrospective nature of the study and biases such as immortal time bias, our data on radiotherapy remain nonetheless interesting, notably showing that eight previously irradiated patients with nonmetastatic relapse underwent re‐irradiation in a partially similar field. Despite lacking a central RT field review, these data emphasize systematically discussing RT, even in previously irradiated patients. The 6‐month PFS rate of 60% for patients with exclusive local/locoregional relapse underscores promptly implementing local treatment in relapse management.

Although the efficacy of additional maintenance therapy in preventing long‐term relapse and enhancing survival has been established in newly diagnosed high‐risk patients with RMS [16], it remains largely unexplored in relapse settings [13, 27, 28]. This assessment is relevant given the substantial incidence of second relapses, as 45.5% of patients achieving a second complete remission experienced subsequent relapses in our cohort. However, the limited size of our cohort did not allow for conclusive results. Consequently, further studies are warranted to validate the value of therapy duration for relapsed RMS.

Our study confirmed the adverse prognostic value of four independent factors: unfavorable primary tumor site, ≥ 4 drug treatment at diagnosis, prior RT, and early relapse [3]. Notably, neither the alveolar histological subtype nor the *FOXO1* fusion transcript significantly increased the death risk in our study, unlike the findings of Chisholm et al. [3]. The unknown fusion transcript status in many patients, combined with the small population size of our cohort (95 patients) compared to 474 patients in the study by Chisholm et al., may explain these discrepancies. Our study also validated the SIOP‐MMT group's nomogram in an independent cohort. As patients enrolled in the RMS 2005 study received chemotherapy regimens different from those in the SIOP‐MMT 84, 89, and 95 trials, the chemotherapy categories used in our analyses differed slightly from those in the study by Chisholm et al. [3]. Due to our limited sample size, we chose to categorize patients into only two groups. We believe this choice did not significantly affect our results, as the type of chemotherapy had a minimal impact in the SIOP‐MMT group's nomogram, except for patients receiving a 2‐drug regimen, which applied to only one patient in our cohort.

In conclusion, our results underscore the need to enhance first‐line treatment for RMS to reduce the risk of relapse as well as to improve treatment approaches at the time of relapse, for which clear guidelines are currently lacking. This emphasizes the necessity for prospective phase I–II trials designed specifically for patients with relapsed RMS, such as the ongoing multi‐arm multi‐stage FaR‐RMS study (NCT04625907) conducted by the EpSSG, aimed at identifying optimal salvage therapy. Furthermore, integrating local treatment data in these studies is crucial for refining salvage strategies. While awaiting more effective combinations, VIT chemotherapy should be considered the gold standard treatment for patients in relapse who have received alkylating agents, as long as they did not relapse or progress during frontline treatment that included irinotecan. Complete local disease control through surgery and/or RT remains crucial for patient salvage. Importantly, re‐irradiation with curative intent is feasible in relapse settings and should be consistently discussed. Additionally, tumor molecular profiling should be performed whenever feasible to identify potential therapeutic targets and improve patient outcomes [17].

## Author Contributions

**François Sevrin:** conceptualization (equal), data curation (lead), formal analysis (equal), investigation (equal), methodology (equal), project administration (equal), visualization (equal), writing – original draft (lead), writing – review and editing (equal). **Emilie Bogart:** formal analysis (equal), methodology (equal), software (equal), writing – review and editing (equal). **Daniel Orbach:** investigation (equal), resources (equal), writing – review and editing (equal). **Marie Cécile Le Deley:** formal analysis (equal), methodology (equal), software (equal), writing – review and editing (equal). **Pablo Berlanga:** investigation (equal), resources (equal), writing – review and editing (equal). **Valérie Bernier:** investigation (equal), resources (equal), writing – review and editing (equal). **Nadège Corradini:** investigation (equal), resources (equal), writing – review and editing (equal). **Cindy Fayard:** investigation (equal), resources (equal), writing – review and editing (equal). **Florent Guerin:** investigation (equal), resources (equal), writing – review and editing (equal). **Sarah Jannier:** investigation (equal), resources (equal), writing – review and editing (equal). **Marie Karanian:** investigation (equal), resources (equal), writing – review and editing (equal). **Stéphanie Proust:** investigation (equal), resources (equal), writing – review and editing (equal). **Angélique Rome:** investigation (equal), resources (equal), writing – review and editing (equal). **Cécile Verité:** investigation (equal), resources (equal), writing – review and editing (equal). **Véronique Minard‐Colin:** investigation (equal), resources (equal), writing – review and editing (equal). **Anne‐Sophie Defachelles:** conceptualization (equal), investigation (equal), project administration (equal), supervision (lead), validation (equal), writing – review and editing (equal).

## Disclosure

The authors have nothing to report.

## Ethics Statement

The current study is based on RMS 2005 trial data re‐use. The RMS 2005 trial (NCT00379457) was approved in France by the ethics committee of Kremlin Bicêtre on 12/01/2005. Written informed consent was obtained from all patients or their parents or guardians if patients were under 18 years of age before enrolment in the trial. The Institutional Review Board of the Oscar Lambret Centre has approved the current study (CEC‐2023‐005) on 16/03/2023 and has confirmed that no ethical approval by an independent ethics committee was required for the current ancillary study of the RMS 2005 trial. The study complies with the Reference Methodology MR004 of the National Commission on Informatics and Freedoms (CNIL, “Commission Nationale Informatique et Liberté”). We have checked that no patient (or no parent or guardian as appropriate) objected to the use of their medical data for research purposes. The study design was performed in accordance with the Declaration of Helsinki.

## Consent

Our manuscript does not contain any individual person's data in any form (including individual details, images or videos). All study participants provided informed consent.

## Conflicts of Interest

Daniel Orbach reports doing consultant work for the French Larotrectinib transparency committee (consultancy agreement signed with the institution). An independent translational research project conducted by him is partially supported by Bayer (investigation‐supported research). He also reports having consultant activity for Lilly, Merk, Bayer Healthcare, Sanofi, Hoffman La Roche, Novartis, and Eusapharm. Veronique Minard‐Colin reports having consultant activity for Roche (AdBoard; no personal benefits). The other authors have no conflicts of interest to disclose.

## Supporting information


**Figure S1.** Consort diagram.

**Figure S2.** Overall survival curve from the date of surgery performed at relapse for patients with nonmetastatic first relapse based on the quality of surgical resection (*n* = 36).

**Figure S3.** Overall survival curve from the date of first relapse for patients who achieved a second complete remission after a nonmetastatic first relapse, based on the administration or non‐administration of maintenance therapy (*n* = 45).

Data S1.

## Data Availability

The datasets generated and/or analyzed during the current study are available from the corresponding author on reasonable request.
