# Supplementary figures and images for: Therapy and Outcomes of Patients with Relapsed Nonmetastatic Rhabdomyosarcoma: A Report from the French Society of Pediatric Oncology Malignant Mesenchymal Tumor Committee
Source: Cancer Med. 2024 Nov 29;13(23):e70420. doi: 10.1002/cam4.70420 (PMC11605160; doi:10.1002/cam4.70420)

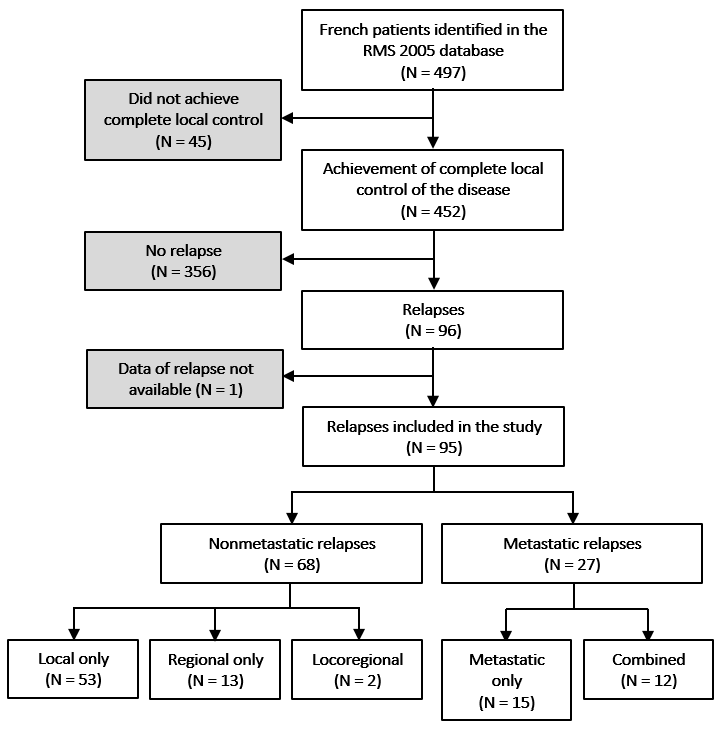

Supplement: Supplementary file 1 — Figure S1. Consort diagram. [file CAM4-13-e70420-s004.docx]

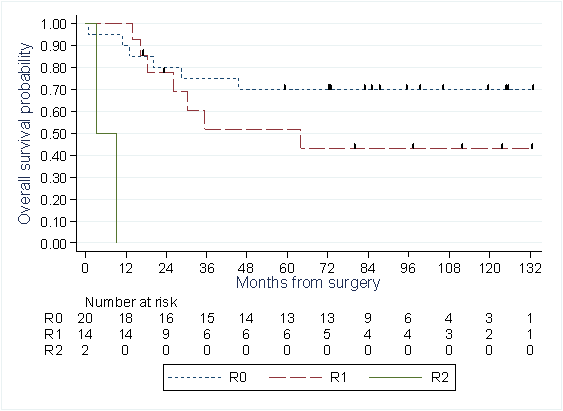

Supplement: Supplementary file 2 — Figure S2. Overall survival curve from the date of surgery performed at relapse for patients with nonmetastatic first relapse based on the quality of surgical resection (n = 36). [file CAM4-13-e70420-s001.docx]

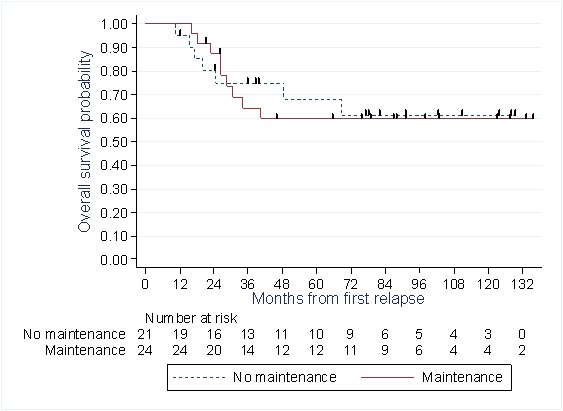

Supplement: Supplementary file 3 — Figure S3. Overall survival curve from the date of first relapse for patients who achieved a second complete remission after a nonmetastatic first relapse, based on the administration or non‐administration of maintenance therapy (n = 45). [file CAM4-13-e70420-s002.docx]
