# Supplementary material for: Therapy and Outcomes of Patients with Relapsed Nonmetastatic Rhabdomyosarcoma: A Report from the French Society of Pediatric Oncology Malignant Mesenchymal Tumor Committee
Source: Cancer Med. 2024 Nov 29;13(23):e70420. doi: 10.1002/cam4.70420 (PMC11605160; doi:10.1002/cam4.70420)
Supplement: Supplementary file 4 — Data S1. [file CAM4-13-e70420-s003.docx]

**METHODS – SUPPLEMENTARY INFORMATION**

*Initial diagnosis and first-line therapy:*

Eligibility criteria for the RMS 2005 study included a histological diagnosis of rhabdomyosarcoma, no evidence of metastatic lesions, age between 6 months and 21 years, no previous treatment except primary surgery, no pre-existing illness preventing treatment, no previous malignancy, an interval between diagnostic surgery and chemotherapy ≤8 weeks, and availability of diagnostic material for central pathology review.

Risk groups and randomization were assigned based on local assessment. Molecular confirmation of FOXO1 presence was advised but not mandatory for classifying a tumor as alveolar. Patients were categorized into specific risk groups using six prognostic factors identified in a common retrospective analysis of European protocols: histological subtype, Intergroup Rhabdomyosarcoma Study (IRS) grouping, tumor primary site and size, nodal involvement, and patient age.

The treatment plans varied according to the assigned risk group (15). First-line chemotherapy regimens included vincristine and d-actinomycin (VA); ifosfamide, vincristine, and d-actinomycin (IVA); ifosfamide, vincristine, d-actinomycin, and doxorubicin (IVADo). Depending on the assigned risk group, patients might also undergo further surgery and/or radiotherapy. Patients at high risk of relapse (those with nonmetastatic incompletely resected embryonal rhabdomyosarcoma occurring at unfavorable sites with age ≥10 years or tumor size >5 cm, or both; those with nonmetastatic rhabdomyosarcoma with nodal involvement; and those with nonmetastatic alveolar rhabdomyosarcoma without nodal involvement) could receive 6-month maintenance therapy with vinorelbine-cyclophosphamide (VC) after randomization (16). Patients with nonmetastatic alveolar rhabdomyosarcoma with nodal involvement systematically received maintenance treatment (14).

*Definition of relapse types:*

Local recurrence referred to a recurrence at the primary site or adjacent tissues, regional recurrence referred to a recurrence involving regional draining lymph nodes, and metastatic recurrence referred to the tumor appearance at distant lymph nodes or other sites.

*Recommendations provided by the EpSSG in 2013 for the management of RMS at first relapse:*

Enrolment in the phase II VIT trial (NCT01355445) was strongly recommended for the first relapse.

Other recommended chemotherapy regimens included:

1. vincristine-irinotecan off study,
2. vincristine-cyclophosphamide-doxorubicin-etoposide,
3. vinorelbine-cyclophosphamide,
4. topotecan-vincristine-doxorubicin.

Recommended duration of treatment was as follows:

1. local recurrence without prior radiotherapy: at least 6 courses of chemotherapy,
2. local recurrence in the radiotherapy field: six courses of chemotherapy + 1 year of maintenance therapy

metastatic recurrence: six courses of chemotherapy + up to 2 years of maintenance therapy.

**Supplementary Table S1. Second-line chemotherapy regimens at first relapse.**

| **Chemotherapy regimen (N=93)** | **Overall** | | **Before 2012**  **(N = 41)** | | **After 2012**  **(N = 52)** | |
| --- | --- | --- | --- | --- | --- | --- |
|  | **N** | **%** | **N** | **%** | **N** | **%** |
| **Anthracycline-based chemotherapy** | 33 | 35.5% | 22 | 53.7% | 11 | 21.2% |
| CEV and IVE alternately | 10 | 10.8% | 8 | 19.5% | 2 | 3.8% |
| Carboplatin and doxorubicin | 8 | 8.6% | 5 | 12.2% | 3 | 5.8% |
| Ifosfamide and doxorubicin | 3 | 3.2% | 2 | 4.9% | 1 | 1.9% |
| Vincristine, irinotecan, and doxorubicin | 3 | 3.2% | 0 | 0% | 3 | 5.8% |
| IVADo | 2 | 2.2% | 2 | 4.9% | 0 | 0% |
| CEV | 1 | 1.1% | 0 | 0% | 1 | 1.9% |
| Cisplatin and doxorubicin | 1 | 1.1% | 1 | 2.4% | 0 | 0% |
| Cyclophosphamide and doxorubicin | 1 | 1.1% | 1 | 2.4% | 0 | 0% |
| Carboplatin, doxorubicin and cyclophosphamide, doxorubicin alternately | 1 | 1.1% | 1 | 2.4% | 0 | 0% |
| Carboplatin, doxorubicin and VINCAEPI alternately | 1 | 1.1% | 1 | 2.4% | 0 | 0% |
| Carboplatin, doxorubicin and vincristine, cyclophosphamide, etoposide  alternately | 1 | 1.1% | 1 | 2.4% | 0 | 0% |
| Vincristine, cyclophosphamide, etoposide, and doxorubicin | 1 | 1.1% | 0 | 0% | 1 | 1.9% |
| **Irinotecan-based chemotherapy** | 41 | 44.1% | 5 | 12.2% | 36 | 69.2% |
| VIT or VI |  |  |  |  |  |  |
| VIT | 21 | 22.6 | 1 | 2.4% | 20 | 38.5% |
| VI | 17 | 18.3 | 3 | 7.3% | 14 | 26.9% |
| Other Irinotecan-based chemotherapy with alkylating agent |  |  |  |  |  |  |
| VAC and VI alternately | 1 | 1.1% | 0 | 0% | 1 | 1.9% |
| VAC and VIT alternately | 1 | 1.1% | 0 | 0% | 1 | 1.9% |
| Other Irinotecan-based chemotherapy without alkylating agent |  |  |  |  |  |  |
| FOLFIRI | 1 | 1.1% | 1 | 2.4% | 0 | 0% |
| **Alkylating agent-based chemotherapy without anthracyclines, platinum compounds and irinotecan** | 4 | 4.3% | 3 | 7.3% | 1 | 1.9% |
| VAC | 2 | 2.2% | 2 | 4.9% | 0 | 0% |
| IVA | 1 | 1.1% | 0 | 0% | 1 | 1.9% |
| VAC and IVE alternately | 1 | 1.1% | 1 | 2.4% | 0 | 0% |
| **Platinum-based chemotherapy** | 7 | 7.5% | 6 | 14.6% | 1 | 1.9% |
| ICE | 2 | 2.2% | 2 | 4.9% | 0 | 0% |
| GEMOX | 2 | 2.2% | 2 | 4.9% | 0 | 0% |
| Topotecan and carboplatin | 1 | 1.1% | 1 | 2.4% | 0 | 0% |
| VINCAETO | 1 | 1.1% | 0 | 0% | 1 | 1.9% |
| Cisplatin and etoposide | 1 | 1.1% | 1 | 2.4% | 0 | 0% |
| **Low-dose chemotherapy** | 8 | 8.6% | 5 | 12.2% | 3 | 5.8% |
| Vinorelbine and cyclophosphamide | 7 | 7.5% | 4 | 9.8% | 3 | 5.8% |
| Cyclophosphamide | 1 | 1.1% | 1 | 2.4% | 0 | 0% |

CEV, carboplatin, epirubicin, vincristine; FOLFIRI, 5FU, irinotecan, leucovorine; GEMOX, gemcitabine, oxaliplatin; ICE, ifosfamide, carboplatin, and etoposide; IVA, ifosfamide, vincristine, and D-actinomycin; IVADo, ifosfamide, vincristine, actinomycin, and doxorubicin; IVE, ifosfamide, vincristine, and etoposide; N, number; VAC, vincristine, D-actinomycin, and cyclophosphamide; VI, vincristine, irinotecan; VINCAETO, vincristine, carboplatin, and etoposide; VIT, vincristine, irinotecan, and temozolomide.

**Supplementary Table S2. Response to second-line chemotherapy after 3 ± 1 courses according to the time interval from diagnosis to relapse (N=82, MD=9).**

|  | **Early relapse (<18 months from initial diagnosis)** | | **Late relapse (≥18 months from initial diagnosis)** | |
| --- | --- | --- | --- | --- |
|  | Response rate (%) | | Response rate (%) | |
| **Anthracycline-based chemotherapy** |  |  |  |  |
| ORR | 7/15 | 47% | 8/14 | 57% |
| SD | 4/15 | 27% | 4/14 | 29% |
| PD | 4/15 | 27% | 2/14 | 14% |
| **Irinotecan-based chemotherapy** |  |  |  |  |
| ORR | 9/15 | 60% | 15/23 | 65% |
| SD | 1/15 | 7% | 7/23 | 30% |
| PD | 5/15 | 33% | 1/23 | 4% |
| ***Including VIT or VI*** |  |  |  |  |
| *ORR* | *8/14* | *57%* | *13/21* | *62%* |
| *SD* | *1/14* | *7%* | *7/21* | *33%* |
| *PD* | *5/14* | *36%* | *1/21* | *5%* |
| **Alkylating agent-based chemotherapy without irinotecan** |  |  |  |  |
| ORR |  |  | 3/3 | - |
| SD |  |  |  |  |
| PD |  |  |  |  |
| **Platinum-based chemotherapy** |  |  |  |  |
| ORR | 1/2 | - | 3/4 | - |
| SD | 1/2 | - | 1/4 | - |
| PD |  |  |  |  |
| **Low-dose chemotherapy** |  |  |  |  |
| ORR | 1/4 | - | 1/2 | - |
| SD | 1/4 | - | 1/2 | - |
| PD | 2/4 | - |  |  |
| **All groups of second-line chemotherapy regimen** |  |  |  |  |
| ORR | 18/36 | 50% | 30/46 | 65% |
| SD | 7/36 | 19% | 13/46 | 28% |
| PD | 11/36 | 31% | 3/46 | 7% |

N, number; MD, missing data; ORR, objective response rate (including complete response and partial response); PD, progressive disease; SD, stable disease; VIT, vincristine, irinotecan, and temozolomide.

**Figure captions**

**Supplementary Fig. S1. Consort diagram.**

**Supplementary Fig. S2. Overall survival curve from the date of surgery performed at relapse for patients with nonmetastatic first relapse based on the quality of surgical resection (N=36).**

The time between the date of relapse and the date of surgery in months based on the quality of surgical resection:

| Quality of surgical resection | Mean | Standard deviation | Min | Max |
| --- | --- | --- | --- | --- |
| R0 (N=20) | 4.2 | 1.9 | 2.0 | 9.6 |
| R1 (N=14) | 3.7 | 2.8 | 0.4 | 12.0 |
| R2 (N=2) | 3.8 | 0.8 | 3.2 | 4.3 |

**Supplementary Fig. S3. Overall survival curve from the date of first relapse for patients who achieved a second complete remission after a nonmetastatic first relapse, based on the administration or non-administration of maintenance therapy (N=45).**
